# Triterpenoid and Steroidal Saponins Differentially Influence Soil Bacterial Genera

**DOI:** 10.3390/plants10102189

**Published:** 2021-10-15

**Authors:** Masaru Nakayasu, Shinichi Yamazaki, Yuichi Aoki, Kazufumi Yazaki, Akifumi Sugiyama

**Affiliations:** 1 Research Institute for Sustainable Humanosphere, Kyoto University, Gokasho, Uji 611-0011, Japan; masaru_nakayasu@rish.kyoto-u.ac.jp (M.N.); yazaki@rish.kyoto-u.ac.jp (K.Y.); 2 Tohoku Medical Megabank Organization, Tohoku University, Sendai 980-8573, Japan; yamazaki.shinichi@megabank.tohoku.ac.jp (S.Y.); aoki@megabank.tohoku.ac.jp (Y.A.)

**Keywords:** bacterial communities, Burkholderiaceae, steroid, oleanane, rhizosphere, saponin, Sphingomonadaceae

## Abstract

Plant specialized metabolites (PSMs) are secreted into the rhizosphere, i.e., the soil zone surrounding the roots of plants. They are often involved in root-associated microbiome assembly, but the association between PSMs and microbiota is not well characterized. Saponins are a group of PSMs widely distributed in angiosperms. In this study, we compared the bacterial communities in field soils treated with the pure compounds of four different saponins. All saponin treatments decreased bacterial α-diversity and caused significant differences in β-diversity when compared with the control. The bacterial taxa depleted by saponin treatments were higher than the ones enriched; two families, Burkholderiaceae and Methylophilaceae, were enriched, while eighteen families were depleted with all saponin treatments. Sphingomonadaceae, which is abundant in the rhizosphere of saponin-producing plants (tomato and soybean), was enriched in soil treated with α-solanine, dioscin, and soyasaponins. α-Solanine and dioscin had a steroid-type aglycone that was found to specifically enrich Geobacteraceae, Lachnospiraceae, and Moraxellaceae, while soyasaponins and glycyrrhizin with an oleanane-type aglycone did not specifically enrich any of the bacterial families. At the bacterial genus level, the steroidal-type and oleanane-type saponins differentially influenced the soil bacterial taxa. Together, these results indicate that there is a relationship between the identities of saponins and their effects on soil bacterial communities.

## 1. Introduction

At least one million diverse plant specialized metabolites (PSMs) [1] exist that have a wide range of bioactivities and contribute to a plant’s ability to adapt to its environment and protect against pathogens and herbivores [2]. PSMs are also secreted into the rhizosphere, i.e., the zone of soil surrounding the roots, which impacts plant responses to nutrient deficiencies and their interactions with soil-borne organisms, such as symbiosis, attraction, and repelling [3,4,5,6]. Metabolites secreted from roots are called root exudates, and these account for up to 40% of the carbon fixed during photosynthesis [7]. Rhizosphere microbiomes assembled by root exudates promote plant growth and help the host plants overcome biotic and abiotic stresses [8,9]. In the last decade, multiple studies have revealed that PSMs are involved in the formation of the rhizosphere and root microbiome [10,11]. PSM-deficient mutants of certain plant species, such as thale cress (*Arabidopsis thaliana*) and maize (*Zea mays*), show that di-, sester-, tri-terpenoids, coumarins, and benzoxazinoids modulate the root-associated microbiome [12,13,14,15,16,17,18]. The treatment of soils with authentic compounds has also helped to reveal the roles of PSMs such as flavonoids (daidzein and quercetin), alkaloids (nicotine and gramine), benzoxazinoid, and opine (santhopine) in modulating the soil microbiome [19,20,21,22].

**Figure 1:**
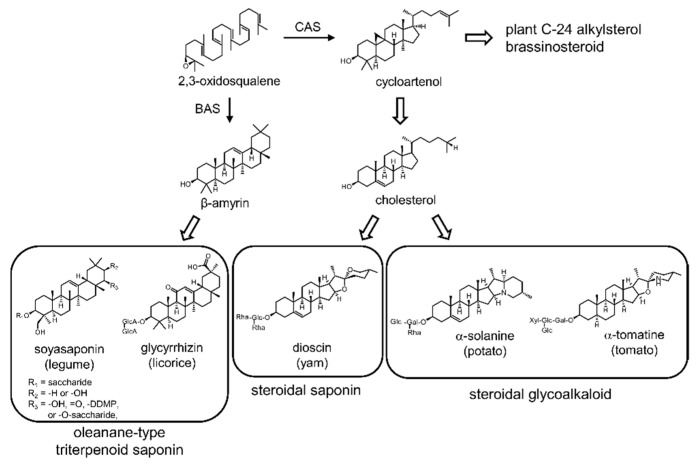
Chemical structures and biosynthesis of the plant saponins tested in this study: BAS, β-amyrin synthase; CAS, cycloartenol synthase.

Saponins are a group of PSMs widely distributed in angiosperm plants. They exhibit biological and pharmacological activities, including antibacterial, antifungal, hemolytic, and cytotoxic properties [23]. They consist of two parts: a hydrophobic skeleton, which is the aglycone unit, and a hydrophilic saccharide, which is the glycosidic unit. Based on aglycone structure, saponins are classified into triterpenoid saponins, steroidal saponins, and steroidal glycoalkaloids (SGAs; Figure 1). Glycyrrhizin is an oleanane-type triterpenoid saponin that is used as a natural sweetener and is found only in licorice (*Glycyrrhiza* spp.) [24]. Soyasaponins are oleanane-type triterpenoid saponins found in several legume plants, including barrel medic (*Medicago truncatula*) and soybean (*Glycine max*). Dioscin is a spirostane-type steroidal saponin used for the semi-synthetic production of pharmaceutical steroidal drugs in *Dioscorea* plants [25]. α-Tomatine and α-solanine are spirosolane- and solanidane-type SGAs in tomato (*Solanum lycopersicum*) and potato (*S. tuberosum*), respectively [26]. All saponins are biosynthesized from 2,3-oxidosqualene—their last common precursor subjected to cyclization, oxidation, and glycosylation [27,28]. This cyclization step catalyzed by oxidosqualene cyclases (OSCs) is the branch point of their biosynthesis. Cyclization to form dammarenediol-II, cucurbitadienol, and β-amyrin leads to triterpenoid saponins, while cyclization to form cycloartenol leads to steroidal saponins and SGAs through cholesterol [28,29,30]. Furthermore, a nitrogen atom is also incorporated into the hydrophobic skeleton during SGA biosynthesis.

Recent studies have shown that saponins are secreted from plant roots into the rhizosphere [31,32,33,34]. Previously, we demonstrated that soyasaponins and α-tomatine were secreted from soybean and tomato roots in both hydroponic and field conditions [35,36,37]. Furthermore, both soyasaponins and α-tomatine were found to alter the bacterial communities and increase Sphingomonadaceae. Soyasaponins enriched *Novosphingobium*, whereas tomatine enriched *Sphingobium* at the genus level [35,37]. Based on these differential effects of the saponins in the soil bacterial communities, we hypothesized a link between saponin identity and soil bacterial community. In this study, we compared bacterial communities in field soils treated with steroidal-type and oleanane-type saponins. We propose that the correlation of rhizosphere microbiome formation with the chemical structure of PSMs may be a factor for microbiome variation among plant species.

## 2. Materials and Methods

### 2.1. Chemicals and Soils

α-Solanine, dioscin, a saponin mixture from soybeans constituted by soyasaponins (mainly group B), and glycyrrhizin were purchased from Extrasynthese (Genay, France), Cayman Chemical (Ann Arbor, MI, USA), FUJIFILM Wako Pure Chemical Corporation (Osaka, Japan), and the Tokyo Chemical Industry Co., Ltd. (Tokyo, Japan), respectively. The soil used was the same as that described in a previous study [20]. Briefly, before plant cultivation, soils were collected from five different points across a field at the Kyoto University of Advanced Science, Kameoka, Kyoto, Japan (34°99′38″ N, 135°55′14″ E), where crops had been cultivated for more than twelve years and soybean had been grown the previous five years. The soils were combined and air-dried in a greenhouse. The soils that passed through a 2 mm sieve were used in the following experiments. Soil chemical and physical properties were measured at the Tokachi Federation of Agricultural Cooperatives [20].

### 2.2. Treatment of Field Soil with Saponins

α-Solanin, dioscin, soyasaponins, and glycyrrhizin were dissolved in methanol and 20, 100, and 500 nmol of each was then dried in 5 mL tubes. Field soil (2 g) and distilled water (600 μL) were added to each tube in biological quadruplicate and then sealed, vortexed, and incubated at 28 °C in the dark. The control was untreated soil that was added to an empty tube. Each soil sample was transferred to a new tube containing the relevant compound once every 3 days over a 15-day incubation period. Tubes were sealed during incubation. After incubation, the tubes were stored at −30 °C until they were used for DNA extraction. The saponin concentrations were determined according to previous works; soyasaponins and α-tomatine in rhizosphere soils of soybean and tomato were approximately 20–80 and 300–1000 nmol g soil^−1^ at the different growth stages, respectively [35,37]. They were equivalent to the low, middle, or high concentrations of saponins treated in this study (i.e., 50, 250, or 1250 nmol g soil^−1^). We previously also treated field soil with a pure compound of α-tomatine in a similar manner to this study [37].

### 2.3. DNA Extraction and 16S rRNA Amplicon Sequencing

DNA was extracted from the saponin-treated soils using a DNeasy PowerSoil Kit (QIAGEN K.K., Tokyo, Japan) and according to the manufacturer’s instructions. The DNA concentrations were quantified using a Qubit dsDNA HS Assay Kit and Qubit 2.0 Fluorometer (Thermo Fisher Scientific, Waltham, MA, USA). The V4 region of the bacterial 16S rRNA was PCR-amplified in technical triplicate using KOD FX Neo (TOYOBO, Osaka, Japan) with the following primer set: 515F (5′-ACACTCTTTCCCTACACGACGCTCTTCCGATCT-GTGCCAGCMGCCGCGGTAA-3′) and 806R (5′-GTGACTGGAGTTCAGACGTGTGCTCTTCCGATCT-GGACTACHVGGGTWTCTAAT-3′) [38]. PCR cycling was carried out at 94 °C for 2 min and 20 cycles at 98 °C for 10 s, 50 °C for 30 s, and 68 °C for 30 s. The PCR products were purified using AMPure XP (Beckman Coulter, Danvers, MA, USA). PCR amplification for attachment of the MiSeq adapters (Illumina, San Diego, CA, USA) was performed with the purified DNA as a template in technical duplicate using KOD FX Neo and primers provided by FASMAC Co. Ltd. (Kanagawa, Japan). PCR cycling was carried out at 94 °C for 2 min and 10 cycles of 98 °C for 10 s, 59 °C for 30 s, and 68 °C for 30 s. The PCR products were purified, and the DNA concentrations were quantified as described above. The purified DNA was mixed in equal amounts and used for 2 × 250-bp paired-end sequencing with MiSeq (Illumina) by FASMAC Co. Ltd. The 16S rRNA amplicon dataset supporting the results of this study has been registered to the DNA Data Bank of Japan (https://www.ddbj.nig.ac.jp, accessed on 13 October 2021) and is to be publicly available (the accession number DRA012729).

### 2.4. Sequence Data Analysis

The acquired sequence data were analyzed using the QIIME2 pipeline (version 2019.7) [39]. The bases, other than the 21st to 200th of the paired-end sequences, were trimmed, and error-corrected amplicon sequence variants (ASVs) were constructed using DADA2 [40] with the q2-dada2 plugin in QIIME2. Multiple alignments of the obtained ASV sequences were created using MAFFT [41], and phylogenetic trees were constructed using FastTree [42] in the q2-phylogeny plugin. Taxonomic assignment of the ASVs was performed using the Naïve Bayes classifier with the Silva rRNA database release 132 [43,44]. ASV dataset with 19,105–65,734 reads per sample was obtained (Table S1). Shannon’s diversity indices as α-diversity and both weighted and unweighted UniFrac distances as β-diversity were calculated from a subsampled ASV dataset with 19,000 sequences per sample using the core-metrics-phylogenetic pipeline in the q2-diversity plugin within QIIME2 (Figure S2). The relative abundances of the bacterial taxa were calculated from the ASV-read abundances.

### 2.5. Statistical Analysis

The Welch’s *t*-test, corrected by the Benjamini–Hochberg method, was carried out using the pairwise.t.test function of R package “stats”. The principal coordinate analysis (PCoA) plots based on the weighted and unweighted UniFrac distances for each saponin-treated soil were generated using the cmdscale function in the R package “stats”. Hierarchical clustering was performed using the complete-linkage method with the hclust function that was also in the R package “stats”. Permutational multivariate analysis of variance (PERMANOVA) was performed using the adonis function in the R package “vegan” [45]. The linear discriminant analysis (LDA) effect size (LEfSe) method [46] was applied using default parameters to detect differentially abundant taxa at the family and genus levels. An adjusted *p* value of <0.05 for Kruskal–Wallis and an LDA score of >2 was used to define significant differences. Low abundant taxa (the mean relative abundance of all samples <0.1%) were filtered out for the LEfSe method.

## 3. Results

### 3.1. Bacterial Diversity in the Saponin-Treated Soils

**Figure 2:**
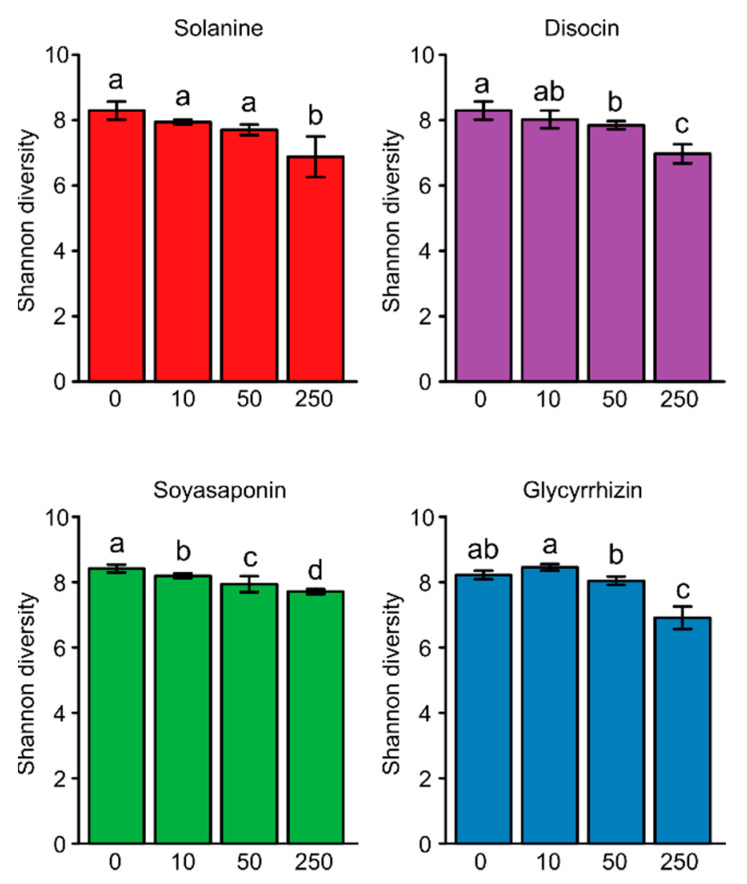
Shannon diversity indices of the bacterial communities in the soils treated with saponins, 10, 50, and 250 nmol (low, middle, and high concentrations) g soil^−1^. Error bars indicate standard deviation (*n* = 4). Different letters (a–d) indicate statistically significant differences (*p* < 0.05) by Welch's *t*-test, corrected by the Benjamini–Hochberg method. Each saponin-0 indicates untreated soils.

**Figure 3:**
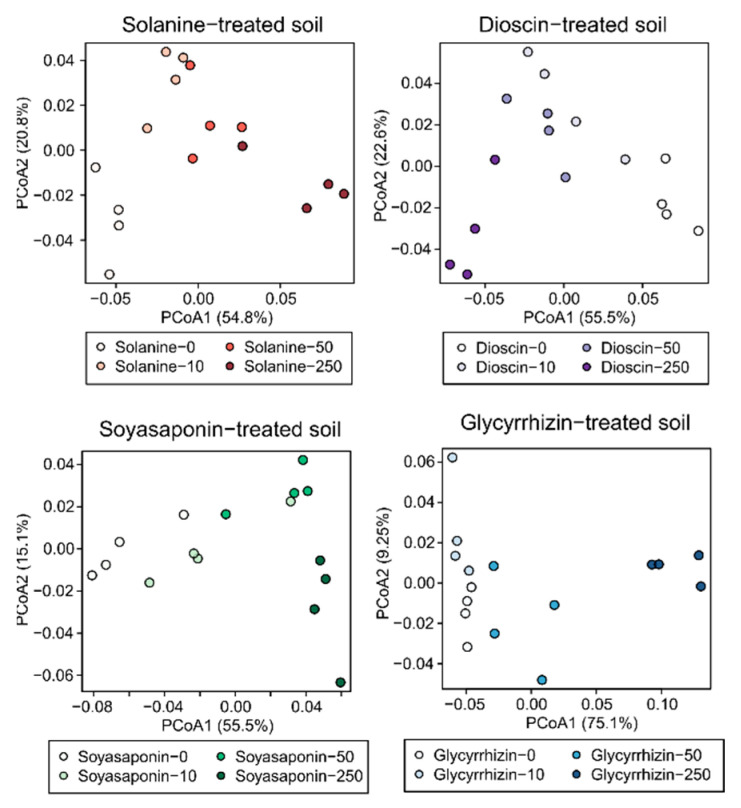
Weighted UniFrac-based principal coordinate analysis (PCoA) of the bacterial communities in the soils treated with saponins, 10, 50, and 250 nmol (low, middle, and high concentrations) g soil^−1^. Each saponin−0 indicates untreated soils.

We treated field soil with α-solanine, dioscin, soyasaponins, and glycyrrhizin compounds at doses of 10, 50, and 250 nmol g soil^−1^. α-Diversity in untreated soils was slightly higher than 8, and the high concentration of each saponin treatment (250 nmol g soil^−1^) significantly reduced the α-diversity compared with control (*p* < 0.05; Figure 2). The PCoA, based on the weighted UniFrac distance metric (β-diversity), showed that the bacterial communities of all saponin-treated soils were distinct from those of the untreated soil (Figure 3). The PERMANOVA analysis confirmed these significant differences (α-solanine, R^2^ = 0.67, *p* < 0.01; dioscin, R^2^ = 0.69, *p* < 0.01, soyasaponins, R^2^ = 0.62, *p* < 0.01, and glycyrrhizin, R^2^ = 0.80, *p* < 0.01). The PCoA, based on the unweighted UniFrac distance metric, also showed their clear distinction (Figure S2 and Table S2). These results indicate that all the saponins tested modified the bacterial communities in the soil.

### 3.2. Effects of the Saponin Treatments on Bacterial Families in the Soil

**Figure 4:**
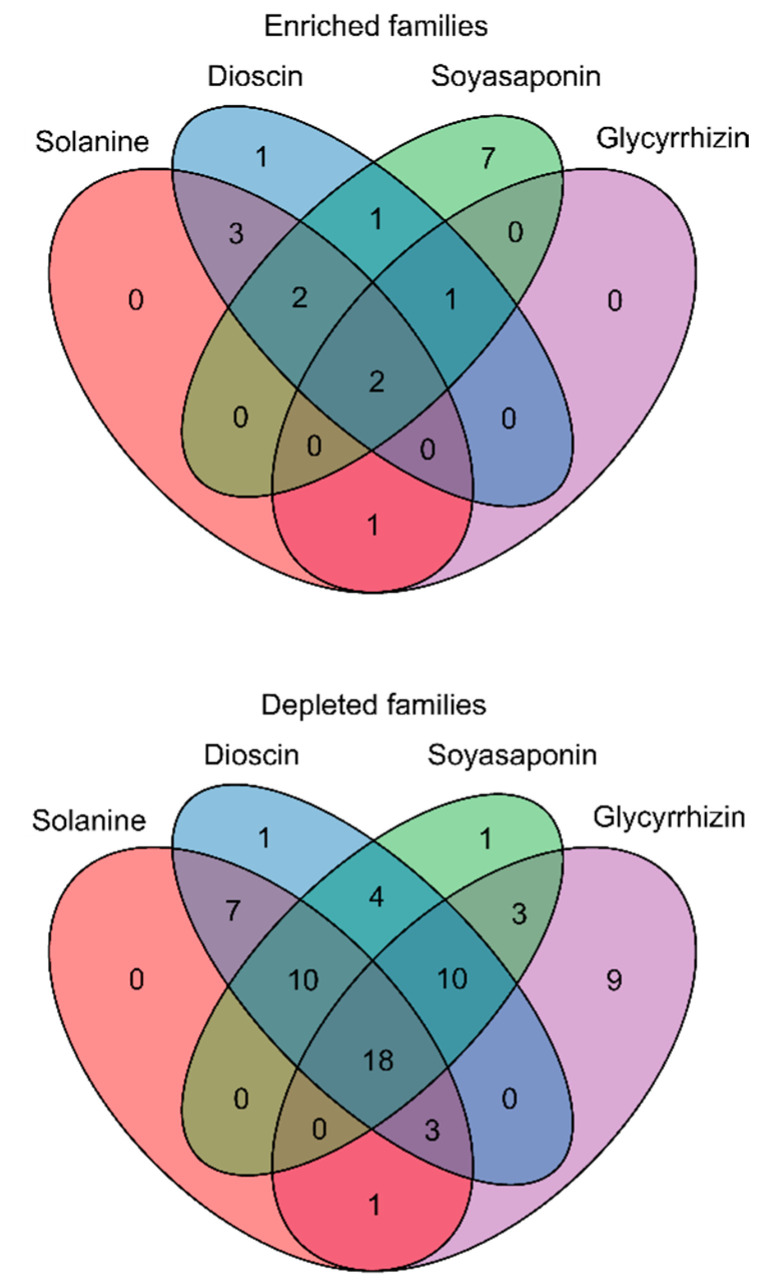
Venn diagrams to illustrate the overlap of the significantly enriched or depleted bacterial families in the soils treated with saponins, 250 nmol (high concentration) g soil^−1^ compared with untreated soils using LEfSe method (an adjusted *p*-value < 0.05). The numbers of bacterial families are shown in the ellipses.

**Figure 5:**
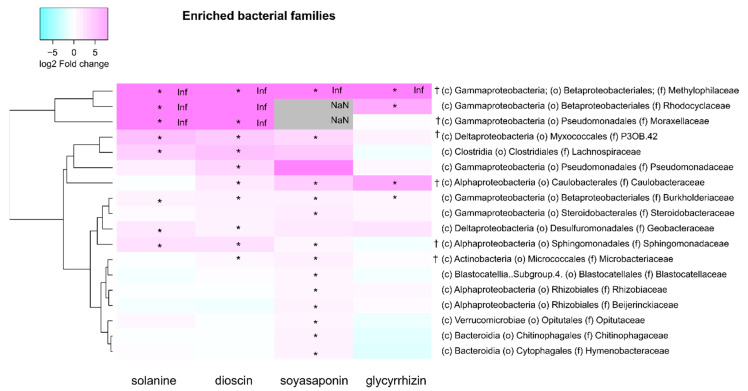
Clustered heatmaps showing the ratio of the relative abundances of the bacterial families (the mean of all samples >0.1%) in the saponin-treated soils to untreated soils. The color of the heatmaps indicates log2 fold changes in the relative abundances. Asterisks indicate statistically significant differences in the soils treated with saponins, 250 nmol (high concentrations) g soil^−1^ using the LEfSe method (an adjusted *p*−value < 0.05). The daggers indicate families significantly enriched in tomatine-treated soil in previous work (Nakayasu et al., 2021). c, class; f, family; o, order; NaN; not a number; Inf, infinity (drawn by substituting eight for log2 fold changes).

We compared the abundance of the bacterial taxa between the saponin-treated and untreated soils at the bacterial family level. The increase and decrease in the relative abundances of taxa in soil samples were defined as “enrichment” and “depletion”, respectively. α-Solanine, dioscin, soyasaponins, and glycyrrhizin significantly enriched 8, 10, 13, and 4 families but depleted 39, 53, 46, and 44 families, respectively (Figure 4 and Table S3). Among these, Burkholderiaceae and Methylophilaceae were enriched in all treatments, and 18 families were depleted in all the treatments (Figure 4 and Figure 5, and Table S3). Except for α-solanine, the saponins enriched Caulobacteraceae, and, except for glycyrrhizin, they enriched P3OB-42 and Sphingomonadaceae (Figure 4 and Figure 5, and Table S3). The α-solanine and dioscin, which have a steroid-type aglycone, specifically enriched Geobacteraceae, Lachnospiraceae, and Moraxellaceae, while soyasaponins and glycyrrhizin, which have an oleanane-type aglycone, did not specifically increase any bacterial family (Figure 4 and Figure 5, and Table S3).

### 3.3. Taxonomic Composition of the Bacterial Families Enriched by the Saponin Treatments

**Figure 6:**
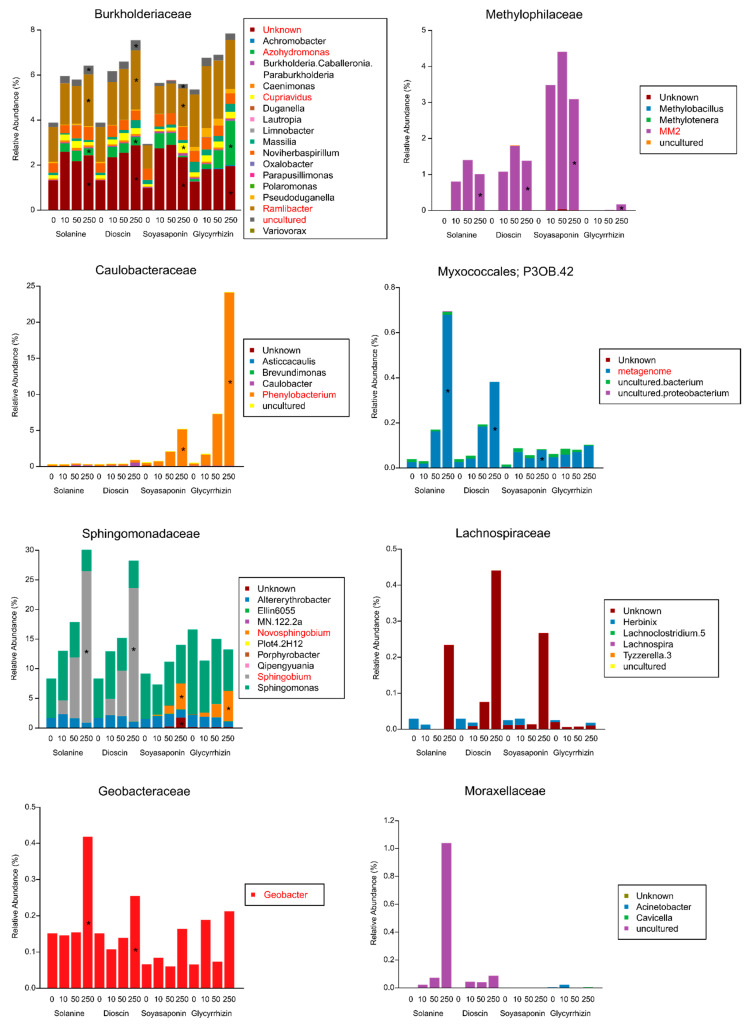
The mean relative abundances for the bacterial genera found in the representative families enriched in the soils treated with saponins, 10, 50, and 250 nmol (low, middle, and high concentrations) g soil^−1^ (*n* = 4). Asterisks and red letters indicate significantly enriched genera (the mean of all samples >0.1%) in soils treated with saponins, 250 nmol (high concentrations) g soil^−1^ using the LEfSe method (an adjusted *p*-value < 0.05).

**Figure 7:**
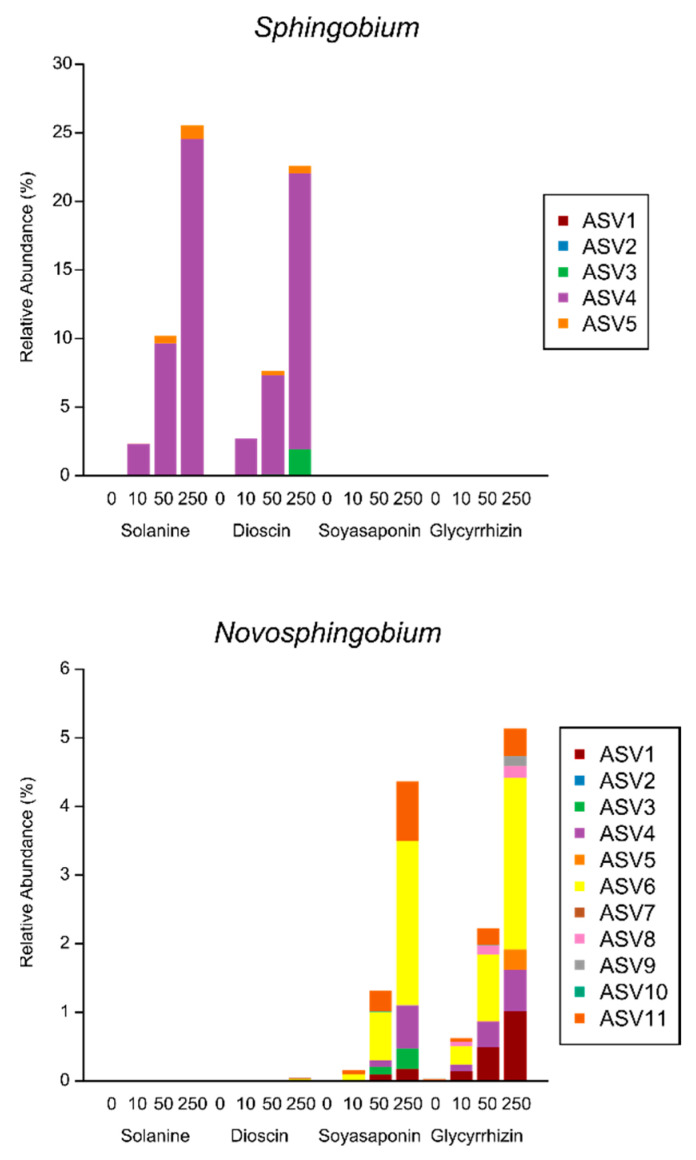
The mean relative abundances of amplicon sequence variants (ASVs) found in the genera *Novosphingobium* and *Sphingobium* of Sphingomonadaceae in soils treated with 10, 50, and 250 nmol (low, middle, and high concentrations) of saponins g soil^−1^ (*n* = 4).

The bacterial families that were significantly enriched by the saponin treatments were further investigated at the genus level. Among the Burkholderiaceae, which were commonly enriched in all the saponin-treated soils, the *Azohydromonas*, *Cupriavidus*, *Ramlibacter*, and uncultured genera were differently altered with the various saponin treatments, while the unknown genus was increased in all the saponin treatments (Figure 6 and Table S4). In contrast to the Burkholderiaceae, a single genus was markedly abundant in Methylophilaceae, Caulobacteraceae, and Geobacteraceae. The *MM2* genus within the Methylophilaceae was dominant in all the saponin-treated soils, but its relative abundance in the glycyrrhizin-treated soils was lower when compared with the other saponins (Figure 6 and Table S4). *Phenylobacterium* was strongly enriched by the oleanane-type saponins (soyasaponins and glycyrrhizin) but not by the steroidal-type saponins (α-solanine and dioscin) in Caulobacteraceae. Among the Geobacteraceae, *Geobacter* was significantly increased only by the steroid-type saponin treatments (Figure 6 and Table S4). *Novosphingobium* was dominant in the treatment with oleanane-type saponins, whereas *Sphingobium* was abundant in the treatment of steroidal-type saponins within the genus belonging to Sphingomonadaceae (Figure 6 and Table S4). At the ASV level of the Sphingomonadaceae, the relative abundances of multiple ASVs were increased in the oleanane-type saponin-treated soils, while that of one ASV (ASV 4) was particularly rich in steroid-type saponin-treated soils (Figure 7 and Table S5), which was the same ASV that was accumulated in α-tomatine- and tomatidine-treated soils and the tomato rhizosphere soil [37].

## 4. Discussion

Treating soil with pure compounds has revealed the strong influences PSMs have in shaping the microbiota [19,20,21,22,35,37,47]. In this study, we treated soil with saponins that had either steroid- or oleanane-type aglycones. The bacterial α-diversity was found to decrease with all four saponin treatments, which was consistent with previous results for benzoxazolin-2(3H)-one (BOA), quercetin, daidzein, flavonoid mixture, α-tomatine, and tomatidine [20,22,37,47]. The decreases found in the bacterial α-diversity were in accordance with previous observations made for the rhizosphere when affected by root exudates [48,49]. In contrast, the treatment of barley indole-alkaloid gramine did not reduce α-diversity, although it did modify the bacterial community structures [19,22]. Intriguingly, the α-diversities in the rhizospheres of the mutants deficient in benzoxazinoids, coumarins, and α-tomatine were comparable with those in wildtypes of maize, thale cress, and tomato, respectively [13,14,37,50,51]. On the other hand, the thale cress triterpenoid mutants harbored root bacterial communities with lower α-diversities than the wildtype [15]. The accumulation of intermediates or the effects of other metabolites on the mutants may alleviate the decrease in the α-diversity by the PSMs.

In accordance with our previous studies with daidzein and α-tomatine [20,37], we found that the bacterial taxa depleted by the saponin treatments were greater than the enriched ones. In the treatments with gramine or quercetin, however, the results were inconsistent [22]. These variations may be caused by differences in the concentrations of the PSMs between our study and that of Schütz et al. [22] (the former: 1250 nmol g soil^−1^ and the latter: 33 nmol g soil^−1^) and the promotion and inhibition effects of the PSMs on the growth of soil microbes. There are some taxa commonly enriched by the PSM treatments and in the host plant rhizosphere and roots, such as: Comamonadaceae and Microbacteriaceae by daidzein and soybean root; *Arthrobacter* of Micrococcaceae by santhopine, nicotine, and tobacco roots; *Sphingobium* of Sphingomonadaceae by α-tomatine, tomatidine, and tomato root; and Caulobacteraceae and *Novosphingobium* of Sphingomonadaceae by soyasaponin Bb and soybean root [20,21,35,47]. It has also been reported that the bacterial communities in the soils treated with daidzein, α-tomatine, nicotine, and santhopine are closer to those of the rhizosphere or endosphere of the respective host plants than the bulk soils, defined as a soil that does not adhere to plant roots [20,21,47]. Rhizosphere and root microbiota have been reported in several saponin-synthesizing plants. Bradyrhizobiaceae, Rhizobiaceae, and Sphingomonadaceae are highly abundant in the taproots of sugar beet (*Beta vulgaris*), which accumulates triterpenoid saponins in its roots when grown in disease-suppressive soils [52,53]. Bacillaceae, Burkholderiaceae, Mycobacteriaceae, Rhizobiaceae, and Sphingomonadaceae are abundant in the roots of Chinese ginseng (*Panax notoginseng*) where ginsenosides are abundant when compared to its leaves and stems [54]. The abundant bacterial families in the rhizosphere and rhizoplane of α-solanine-producing potato are composed of Sphingomonadaceae, Flavobacteriaceae, Pseudomonadaceae, Sphingobacteriaceae, Oxalobacteraceae, Moraxellaceae, and Comamonadaceae [55]. Comamonadaceae is assigned to Burkholderiaceae when the Silva rRNA database release 132 is used as a taxonomy classifier. It is to be noted that identical sequences can be annotated to different taxonomy, depending on the database. Burkholderiaceae has also been identified as a predominant member in the phyllosphere of a yam species (*D. bulbifera*) [56], which contains several steroidal saponins, including dioscin [57]. *Streptomyces* and *Sphingobium* are dominant in the root-associated compartments of switchgrass (*Panicum virgatum*)-containing steroidal saponins [58,59]. To the best of our knowledge, there have only been a few microbiome analyses of *Glycyrrhiza* plants outside of the culture-dependent identification of their endophytic bacteria [60]. Consistent with these findings, all four saponins analyzed in this study enriched Burkholderiaceae, and, except for glycyrrhizin, the other three saponins enriched Sphingomonadaceae. Among the Sphingomonadaceae, *Sphingobium* and *Novosphingobium* were specifically enriched by the steroid- and oleanane-type saponin treatments, respectively. Moraxellaceae, which was enriched in the potato rhizoplane [55], was only increased in steroid-type saponin-treated soils. Therefore, our results partially reflect the rhizosphere microbiome of field-grown plants. Confirmation of saponin secretion from diverse plants and analysis of saponin-producing plants’ rhizosphere microbiome will reinforce our findings.

Some bacteria that can survive in the presence of saponins with anti-microbial activities are thought to have resistance to them or the ability to degrade or assimilate them. Metagenome analysis of the steroid degradation pathway from diverse environments revealed that Alphaproteobacteria and Actinobacteria were predominant in the rhizosphere and that most of the Alphaproteobacteria were Sphingomonadaceae and Rhizobiales [61]. In accordance with this, it has been reported that the *Sphingobium* of Sphingomonadaceae that were isolated from α-tomatine-treated soil degraded α-tomatine and tomatidine, and that *Nocardia* and *Arthrobacter* of Actinobacteria modified tomatidine [37,62,63]. Some genera, including *Novosphingobium* of Sphingomonadaceae, *Caulobacter*, and *Burkholderia*, displayed β-glucosidase activities toward ginsenoside [64,65,66]. *Phenylobacterium*, *Amycolatopsis*, *Sediminibacterium*, and *Ochrobactrum* were the predominant taxa correlated with tea saponin degradation in the gut of the camellia weevil [67]. However, bacteria capable of metabolizing aglycones were not isolated from soils, and the saponin catabolic pathway in bacteria remains unclear, especially in the rhizosphere [34]. To elucidate the saponin metabolism and its significance in microbiome formation, it is necessary to isolate bacterial strains belonging to taxa enriched by saponin treatments and to investigate their abilities to assimilate saponins and colonize the rhizosphere of saponin-producing plants.

Rhizosphere and root microbiomes modified by PSMs can be beneficial for plant growth and defense. For example, in maize, soil microbial communities assembled by benzoxazinoids suppressed herbivore growth in the next generation of plants and Oxalobacteraceae enriched by flavones promoted plant growth and nitrogen acquisition [9,14]. Members of the family Burkholderiaceae are reportedly involved in plant–pathogen suppression via the upregulation of induced systemic resistance-associated genes and the production of sulfurous volatile compounds and siderophores [68,69,70]. Members of Sphingomonadaceae have been found to promote plant growth via phytohormone production, alleviation of heavy metal toxicity and drought stress, and pathogen suppression [71,72,73,74,75,76,77]. It is plausible that saponin-producing plants may benefit from attracting those bacterial families to their rhizospheres and roots. A better understanding of interactions between plant and rhizosphere microbiota mediated by saponins would contribute to the elucidation of the mechanisms by which plants shape their microbiota and the effects of root and rhizosphere microbiota on plant growth and health. PSMs, including saponins, have the potential to be used as biostimulants to manipulate the rhizosphere microbiome for improving crop growth and yield. Knowledge of the association between PSMs and the microbiome would benefit us in optimally designing crop rotations in the field.

## Data Availability

All data are available in the paper.

## Supplementary Materials

The following are available online at https://www.mdpi.com/article/10.3390/plants10102189/s1: Figure S1, Rarefaction curves for observed ASVs richness of saponin-treated soils; Figure S2, Unweighted UniFrac-based principal coordinate analysis (PCoA) of the bacterial communities in the soils treated with saponins, 10, 50, and 250 nmol (low, middle, and high concentrations) g soil^−1^; Table S1, Sequencing depth for each sample analyzed to assess the bacterial communities; Table S2, Results of Permutational multivariate analysis of variance (PERMANOVA) in Figure S2; Table S3, Significantly enriched or depleted bacterial families in the soils treated with the saponins, 250 nmol (high concentration) g soil^−1^ when compared with the control soil; Table S4, Significantly enriched or depleted bacterial genera in the soils treated with the saponins, 250 nmol (high concentration) g soil^−1^ when compared with control soil; Table S5, DNA sequences of the amplicon sequence variants (ASVs) detected in the *Novosphingobium* and *Sphingobium* samples analyzed for bacterial communities.
