# Supplementary material for: Triterpenoid and Steroidal Saponins Differentially Influence Soil Bacterial Genera
Source: Plants (Basel). 2021 Oct 15;10(10):2189. doi: 10.3390/plants10102189 (PMC8538258; doi:10.3390/plants10102189)
Supplement: Supplementary file 1 [file plants-10-02189-s001.zip › Figure S1.pdf]

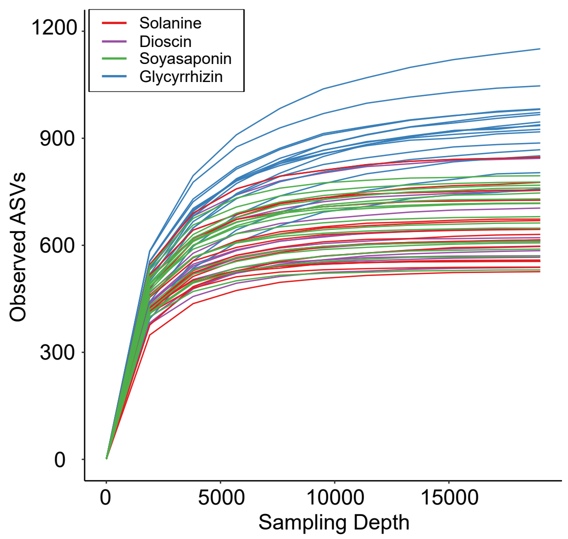

Figure S1. Rarefaction curves for observed ASVs richness of saponin-treated soils. ASVs, amplicon sequence variants.
