# Supplementary material for: Triterpenoid and Steroidal Saponins Differentially Influence Soil Bacterial Genera
Source: Plants (Basel). 2021 Oct 15;10(10):2189. doi: 10.3390/plants10102189 (PMC8538258; doi:10.3390/plants10102189)
Supplement: Supplementary file 1 [file plants-10-02189-s001.zip › Figure S2.pdf]

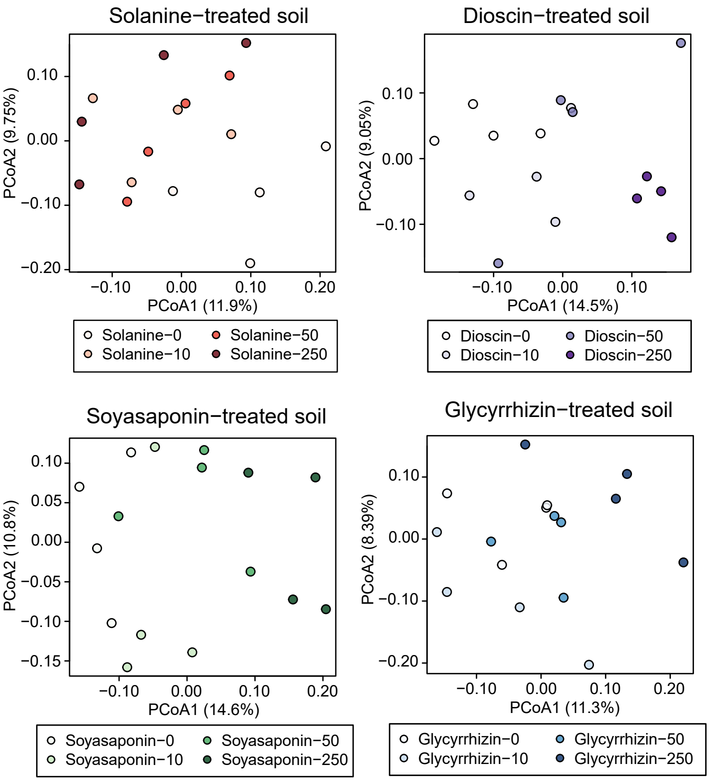

Figure S2. Unweighted UniFrac-based principal coordinate analysis (PCoA) of the bacterial communities in the soils treated with saponins, 10, 50, and 250 nmol (low, middle, and high concentrations) g soil^{-1}.
